# Predicting efficacy of combined assessment with fragmented QRS and severely depressed heart rate variability on outcome of patients with acute myocardial infarction

**DOI:** 10.1007/s00380-021-01930-y

**Published:** 2021-08-23

**Authors:** Yanling Xu, Yijun Yu, Li He, Yuting Wang, Ye Gu

**Affiliations:** Department of Cardiology, Wuhan Fourth Hospital; Puai Hospital affiliated to Tongji Medical College, Huazhong University of Science and Technology, HanZheng Street 473#, QiaoKou District, Wuhan, 430033 China

**Keywords:** Acute myocardial infarction, Fragmented QRS, Heart rate variability, Major adverse cardiovascular events

## Abstract

The association between fragmented QRS (fQRS) and autonomic nervous dysfunction, and major adverse cardiovascular events (MACE) is not fully clear in patients with acute myocardial infarction (AMI). This study aimed to observe whether combined assessment with fQRS and cardiac autonomic nervous function could enhance the predicting efficacy on outcome in AMI patients. A total of 153 consecutive hospitalized AMI patients were included in this retrospective study. Patients were divided into non-fQRS (nfQRS) group and fQRS group according to 12-lead electrocardiogram, into sHRV [severely depressed heart rate variability (HRV): standard deviation of NN intervals (SDNN) < 100 ms and very low frequency (VLF) < 26.7 ms] group and nsHRV (non-severely depressed HRV) group according to 24 h Holter monitoring, and into non-MACE (nMACE) group and MACE group according to 12 months’ follow-up results. The incidence of sHRV was significantly higher in the fQRS group than in the nfQRS group (71.9 vs. 39.3%, *p* < 0.05). The incidences of MACE were 7.4, 22.2, 25.7 and 56.5%, respectively, in nsHRV + nfQRS group, nsHRV + fQRS group, sHRV + nfQRS group and sHRV + fQRS group (*p* < 0.05). Multivariable Cox regression analysis showed that patients in the sHRV + fQRS group had a sixfold higher risk of MACE compared to patients in the nsHRV + nfQRS group (HR = 6.228, 95% CI 1.849–20.984, *p* = 0.003). The predicting sensitivity and specificity on MACE were 81.4 and 58.2% by sHRV, 69.8 and 69.1% by fQRS in these AMI patients. The specificity (81.8%) was the highest with the combination of sHRV and fQRS. Adding sHRV and fQRS to clinical data offered incremental prognostic value. Present results indicate that fQRS is closely related to sHRV, suggesting significant impairment of sympathetic nerve function in AMI patients with fQRS. Combined assessment with fQRS and sHRV enhances the predicting efficacy on outcome in AMI patients.

**Supplementary Information:**

The online version contains supplementary material available at 10.1007/s00380-021-01930-y.

## Introduction

Acute myocardial infarction (AMI) is the main pathogeny of sudden cardiac death in patients with ischemic cardiovascular diseases. Clinical adverse cardiovascular events are not rare despite successful percutaneous coronary intervention (PCI) in AMI patients [1]. Therefore, risk stratification among AMI patients is of crucial importance to define high-risk patients and make individualized decision-making aiming to improve the outcome of AMI patients. Fragmented QRS (fQRS) on electrocardiogram (ECG) is a response to abnormal ventricular myoelectric activity after AMI [2]. The presence of fQRS might indicate severe myocardial insult in AMI patients [3]. Previous studies demonstrated that fQRS was related to higher major adverse cardiovascular events (MACE) in patients with AMI [4–8]. Cardiac autonomic nerve function could be non-invasively assessed by heart rate variability (HRV) [9]. Significantly reduced HRV was evidenced and related to worse outcome in AMI patients [10–14]. Till now, the association between fQRS and autonomic nervous dysfunction is not fully clear in patients with AMI. The present study observed the correlation between fQRS and cardiac autonomic nervous dysfunction, and explored the predicting efficacy of combined assessment with fQRS and sHRV on outcome in AMI patients.

## Materials and methods

### Study population

A total of 156 consecutive hospitalized AMI patients, who were hospitalized in our department from January 2017 to December 2018, were included in this retrospective study. Three patients were lost to follow-up and data from 153 patients were analyzed in this study. Patients were divided into non-fQRS group (nfQRS group, *n* = 89) and fQRS group (*n* = 64) according to the 12-lead ECG at admission, into sHRV [severely depressed HRV: standard deviation of NN intervals (SDNN) < 100 ms [15] and very low frequency (VLF)  < 26.7 ms, *n* = 81] group, and nsHRV (non-severely depressed HRV, n = 72) group according to 24 h Holter monitoring. Patients were also divided into non-MACE (nMACE) group (*n* = 110) and MACE group (*n* = 43) according to the 12 months’ follow-up results. Patients with cardiomyopathy, congenital heart disease, organic heart valvular disease, electrolyte disturbance, bundle branch block, Wolf–Parkinson–White syndrome, atrial fibrillation, pacemaker implantation, malignant tumor and digitalis medication were excluded. This study protocol was approved by the ethical committees of Wuhan Fourth Hospital, Puai Hospital affiliated to Tongji Medical College, Huazhong University of Science and Technology and conducted in compliance with the ethical principles of the Declaration of Helsinki. 24 h Holter monitoring results were obtained in the clinical medical records in this study and the requirement to obtain informed consent was waived by the ethical committees of Wuhan Fourth Hospital, Puai Hospital affiliated to Tongji Medical College, Huazhong University of Science and Technology.

### ECG criteria for fQRS

**Fig. 1:**
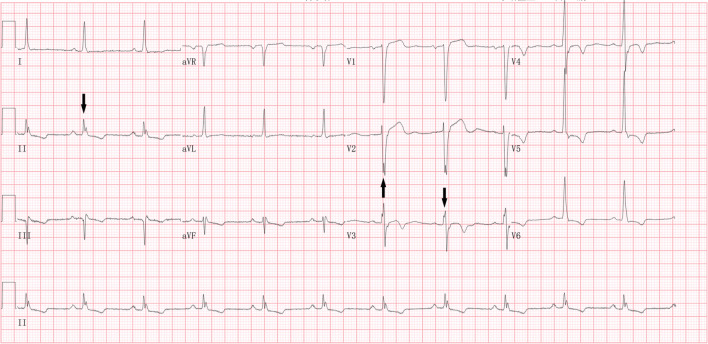
Example of fQRS on a 12-lead ECG (black arrow). *ECG* electrocardiogram, *fQRS* fragmented QRS

fQRS was first proposed by Das in 2006 [16]. The RSR pattern included the QRS interval (QRS duration  < 120 ms) with or without the Q wave. It was defined by the presence of an additional R wave (R’) or notching in the nadir of the S wave, or the presence of R’ (fragmentation) in two contiguous leads, corresponding to a major coronary artery territory [16] (Fig. 1). The fQRS was recorded by 12-lead ECG (GE, Marquette, model Mac 800, filter range, 0.04–150 Hz, AC filter, 80 Hz, 25 mm/s, 10 mm/mV, General Electric Company, Boston, USA) and analyzed by two independent ECG diagnostician blinded to AMI patients.

### HRV analysis

All patients received 24 h Holter monitoring (MARS Software and Seer Light recording box, General Electric Company, Boston, USA) within one week after infarction. 24 h mean heart rate and HRV parameters were analyzed according to the guidelines of the European Society of Cardiology and the North American Society of Pacing and Electrophysiology [17]. The main parameters of time domain are SDNN, standard deviation of all 5 min average NN intervals (SDANN), and square root of mean of the sum of squares of successive NN interval differences (rMSSD), number of successive NN interval differing by  > 50 ms divided by the total number of successive NN intervals (pNN50). The frequency domain parameters are VLF at frequency between 0.0033 and 0.04 Hz, low frequency (LF) at frequency between 0.04 and 0.15 Hz, high frequency (HF) at frequency between 0.15 and 0.4 Hz, and ratio of low frequency/high frequency (LF/HF). Patients were classified according to the combination of time domain index SDNN and frequency domain index VLF. The sHRV was defined as both SDNN  < 100 ms [15] and VLF  < 26.7 ms (the optimal cutoff value identified by the Youden’s index from the ROC curve), and other patients were nsHRV.

### Follow-up

All patients were followed up for 12 months by clinic visit or phone call. The main endpoint was MACE, which included all-cause mortality, non-fatal reinfarction, non-fatal stroke, heart failure, and urgent revascularization for unstable angina [18].

### Statistical analysis

Kolmogorov–Smirnov test was used for normal distribution of all continuous variables. Continuous data with normal distribution were expressed as mean ± standard deviation and assessed by Student’s *t* test. Non-normal distribution data were expressed as median (inner-quartile distance) and assessed by two-tailed Mann–Whitney *U* test. Categorical variables were expressed as count and percentages, and compared using Chi-square test or Fischer’s exact test, as appropriate. Spearman correlation analysis was performed between fQRS and HRV parameters in the AMI patients. The cutoff value of VLF was derived from receiver operating characteristic curves (ROC) analysis by maximizing the sum of the sensitivity and specificity. The rates of MACE were compared by log-rank test of Kaplan–Meier curve. The hazard ratios (HRs) with 95% confidence intervals (CIs) of MACE were assessed by univariable and multivariable Cox proportional hazards regression analysis. Multivariable Cox regression models adjusting for clinical confounders with backward stepwise method (likelihood ratio) were used. Clinical confounders were identified as basic parameters (including age and sex) and parameters significantly associated all with fQRS, sHRV and MACE [including N-terminal pro-brain natriuretic peptide (NT-proBNP), serum creatinine (Scr) and left ventricular ejection fraction (LVEF)]. NT-proBNP was normalized prior to regression analysis using natural logarithm values (Ln). Incremental model performance was assessed by changes in the Chi-square value for the regression models with enter method. Statistical analyses were performed using IBM SPSS, version 22.0 for Windows (IBM Corp, New York, USA), with *p* < 0.05 (two-tailed test) as statistical significance.

## Results

### Clinical features of AMI patients in nfQRS group and fQRS group

Table 1 showed the clinical characteristics between nfQRS group and fQRS group. The proportion of patients with coronary artery lesion  ≥ 3 and the blood biochemical indexes, such as cardiac troponin I (cTnI), creatine kinase (CK), creatine kinase isoenzyme-MB (CK-MB), total cholesterol (T-Chol), triglyceride (TG), high-density lipoprotein cholesterol (HDL-c), low-density lipoprotein cholesterol (LDL-c), Scr and NT-proBNP, were significantly higher in the fQRS group than in the nfQRS group (all *p* < 0.05). LVEF, SDNN, SDANN, VLF, LF and LF/HF values were significantly lower in the fQRS group than in the nfQRS group (*p* < 0.05). The percentage of sHRV was significantly higher in the fQRS group than in the nfQRS group (71.9 vs. 39.3%, *p* < 0.05).

**Table 1:** Clinical characteristic of nfQRS / fQRS groups and nsHRV/sHRV groups in AMI patients

	nfQRS group (*n* = 89)	fQRS group (*n* = 64)	nsHRV group (*n* = 72)	sHRV group (*n* = 81)
Age (yr)	64.3 ± 10.6	67.4 ± 10.3	62.1 ± 10.7	68.7 ± 9.5**
Male (*n*, %)	62/89 (69.7%)	50/64 (78.1%)	60/72 (83.3%)	52/81 (64.2%)**
Hypertension (*n*, %)	59/89 (66.7%)	46/64 (72.7%)	41/72 (56.9%)	64/81 (79.0%)**
Diabetes mellitus (*n*, %)	23/89 (26.7%)	18/64 (28.8%)	14/72 (19.4%)	27/81 (33.3%)
Cerebral infarction (*n*, %)	14/89 (16.7%)	10/64 (15.2%)	6/72 (8.2%)	18/81 (22.2%)**
STEMI (*n*, %)	34/89 (37.8%)	3/64 (53.0%)	36/72 (50.0%)	32/81 (39.5%)
Infarct site (anterior wall)	59/89 (66.3%)	48/64 (75.0%)	49/72 (68.1%)	58/81 (71.6%)
Number of vascular lesions (≥ 3)	37/89 (41.6%)	46/64 (72.9%)*	36/72 (50.0%)	47/81 (58.0%)
cTnI (ng/mL)	0.52 (2.31)	9.08 (34.43)*	1.90 (11.29)	1.18 (9.78)
CK (U/L)	127 (109)	336 (393)*	178 (321)	199 (268)
CK-MB (U/L)	17 (15)	32.50 (48)*	22 (35)	23 (22)
T-Chol (mmol/L)	4.14 ± 0.94	4.82 ± 1.20*	4.32 ± 1.12	4.52 ± 1.09
TG (mmol/L)	1.36 (0.88)	1.80 (1.04)*	1.67 (1.17)	1.59 (0.81)
HDL-c (mmol/L)	0.99 (0.35)	0.82 (0.25)*	0.91 (0.33)	0.90 (0.31)
LDL-c (mmol/L)	2.47 ± 0.85	2.98 ± 0.93*	2.52 ± 0.93	2.82 ± 0.89**
ALT (U/L)	21 (17)	23 (16)	21.50 (18)	21 (16)
AST (U/L)	27 (18)	30 (38)	29.50 (40)	29 (15)
Scr (μmol/L)	72 (30)	88.05 (35)*	73 (26)	83.40 (39)**
NT-proBNP (pg/mL)	161.70 (460)	1162.75 (4410)*	148.05 (435)	884.20 (2735)**
LVEF (%)	59 (5)	53 (11)*	58 (7)	56 (10)**
24 h mean heart rate (bpm)	66 (13)	68 (14)	64 (10)	72 (16)**
SDNN (ms)	97.82 ± 28.21	72.44 ± 23.43*	108.04 ± 24.42	68.68 ± 18.44**
SDANN (ms)	78.64 ± 26.69	59.41 ± 22.82*	85.97 ± 26.89	56.93 ± 17.95**
rMSSD (ms)	26 (12)	24.50 (14)	30.50 (16)	23 (12)**
pNN50 (%)	5.60 (9)	4.60 (7.50)	7.65 (11.60)	3.20 (5.10)**
VLF (ms)	29.01 ± 12.92	20.80 ± 9.58*	33.34 (10.25)	17.78 (8.18)**
LF (ms)	14.70 (10.34)	10.62 (8.42)*	17.76 (8.20)	9.66 (5.23)**
HF (ms)	10.19 (6.12)	9.42 (5.85)	12.37 (6.19)	8.82 (4.88)**
LF/HF	1.43 ± 0.40	1.16 ± 0.40*	1.49 ± 0.40	1.17 ± 0.38**
sHRV (*n*, %)	35/89 (39.3%)	46/64 (71.9%)*	–	–
fQRS (*n*, %)	–	–	18/72 (25.0%)	46/81 (56.8%)**

*AMI* acute myocardial infarction, *ALT* alanine aminotransferase, *AST* aspartate aminotransferase, *CK* creatine kinase, *CK-MB* creatine kinase isoenzyme-MB, *cTnI* cardiac troponin I, *fQRS* fragmented QRS, *HDL-c* high-density lipoprotein cholesterol, *HF* high frequency, *HRV* heart rate variability, *LDL-c* low-density lipoprotein cholesterol, *LF* low frequency, *LF/HF* ratio of low frequency to high frequency LVEF, left ventricular ejection fraction, *nfQRS* non-fragmented QRS, *nsHRV* non-severely depressed heart rate variability, *NT-proBNP* N-terminal pro-brain natriuretic peptide, pNN50, number of successive NN intervals differing by  > 50 ms divided by the total number of successive NN intervals, rMSSD, square root of mean of the sum of squares of successive NN interval differences, *Scr* serum creatinine, *SDANN* standard deviation of all 5 min average NN intervals, *SDNN* standard deviation of NN intervals, *sHRV* severely depressed heart rate variability, *STEMI* ST-segment elevation myocardial infarction, *T-Chol* total cholesterol, *TG* triglyceride, *VLF* very low frequency

^*^p < 0.05 fQRS group vs. nfQRS group

**p < 0.05 sHRV group vs. nsHRV group

### Clinical features of AMI patients in nsHRV group and sHRV group

Age, history of hypertension and cerebral infarction, LDL-c, Scr, NT-proBNP and 24 h mean heart rate were significantly higher in the sHRV group than in the nsHRV group (all *p* < 0.05). Percentages of male, LVEF, SDNN, SDANN, rMSSD, pNN50, VLF, LF, HF and LF/HF values were significantly lower in the sHRV group than in the nsHRV group (all *p* < 0.05). The percentage of fQRS was significantly higher in the sHRV group compared to the nsHRV group (56.8 vs. 25.0%, *p* < 0.05) (Table 1).

### Spearman correlation of fQRS and HRV parameters in AMI patients

Spearman correlation analysis showed that SDNN, SDANN, VLF, LF and LF/HF values were negatively correlated with fQRS, and sHRV was positively correlated with fQRS in AMI patients (*r* = 0.322, *p* < 0.001) (Table 2).

**Table 2:** Spearman correlation analysis of fQRS and HRV parameters in AMI patients

	*r* value	*p* value
SDNN (ms)	− 0.417	< 0.001
SDANN (ms)	− 0.338	< 0.001
rMSSD (ms)	− 0.076	0.352
pNN50 (ms)	− 0.062	0.445
VLF (ms)	− 0.315	< 0.001
LF (ms)	− 0.293	< 0.001
HF (ms)	− 0.117	0.148
LF/HF	− 0.323	< 0.001
sHRV	0.322	< 0.001

*AMI* acute myocardial infarction, *fQRS* fragmented QRS, *HF* high frequency, *HRV* heart rate variability, *LF* low frequency, *LF/HF* ratio of low frequency to high frequency, pNN50 number of successive NN intervals differing by  > 50 ms divided by the total number of successive NN intervals, *rMSSD* square root of mean of the sum of squares of successive NN interval differences, *SDANN* standard deviation of all 5 min average NN intervals, *SDNN* standard deviation of NN intervals, *sHRV* severely depressed heart rate variability, *VLF* very low frequency

### Follow-up results

Three patients were lost to follow-up, 1 in the nfQRS group and 2 in the fQRS group. There were 13 MACE in the nfQRS group: all-cause mortality (*n* = 2, both were cardiac mortality), re-hospitalization due to heart failure (*n* = 5), non-fatal reinfarction (*n* = 2), urgent revascularization for unstable angina (*n* = 1) and stroke (*n* = 3). There were 30 MACEs in the fQRS group: all-cause mortality (*n* = 5, all were cardiac mortality), rehospitalization due to heart failure (*n* = 8), non-fatal reinfarction (*n* = 5), urgent revascularization for unstable angina (*n* = 4) and stroke (*n* = 8).

According to the HRV classification, 1 patient in the nsHRV group and 2 patients in the sHRV group were lost to follow-up. There were 8 MACEs in the nsHRV group: all-cause mortality (*n* = 1, cardiac mortality), rehospitalization due to non-fatal reinfarction (*n* = 2), urgent revascularization for unstable angina (*n* = 1) and stroke (*n* = 4). There were 35 MACEs in the sHRV group: all-cause mortality (*n* = 6, all were cardiac mortality), rehospitalization due to heart failure (*n* = 13), non-fatal reinfarction (*n* = 5), urgent revascularization for unstable angina (*n* = 4) and stroke (*n* = 7).

### Predicting value of different SDNN for MACE in AMI patients

The predicting values of different SDNN for MACE in AMI patients were as follows: the sensitivity, specificity, positive predictive value and negative predictive value of SDNN  < 100 ms were 86.1, 36.4, 34.6 and 87.0%, respectively, and that of SDNN < 80 ms were 65.1, 66.4, 43.1 and 83.0%, respectively, and that of SDNN < 70 ms were 44.2, 78.2, 44.2, 78.2%, respectively. The sensitivity and negative predictive values of SDNN < 100 ms as cutoff value were the highest (Supplementary Table 1).

### Risk factors of MACE in AMI patients

Age, history of hypertension and cerebral infarction, Scr, NT-proBNP and 24 h mean heart rate were significantly higher in the MACE group than in the nMACE group (*p* < 0.05). Percentages of STEMI and LVEF were significantly lower in the MACE group than in the nMACE group (*p* < 0.05). The SDNN, SDANN, LF, VLF, LF/HF values were significantly lower in the MACE group compared to the nMACE group (*p* < 0.05). The percentages of sHRV (81.4 vs. 41.8%) and fQRS (69.8 vs. 30.9%) were significantly higher in the MACE group compared to the nMACE group (both *p* < 0.001) (Table 3).

**Table 3:** Clinical characteristic of nMACE group and MACE group in AMI patients

	nMACE group (*n* = 110)	MACE group (*n* = 43)	*p* value
Age (yr)	62.3 ± 9.5	74.1 ± 8.3	< 0.001
Male (*n*, %)	84/110 (76.4%)	28/43 (65.1%)	0.158
Hypertension (*n*, %)	66/110 (60.0%)	39/43 (90.7%)	< 0.001
Diabetes mellitus (*n*, %)	26/110 (23.6%)	15/43 (34.9%)	0.158
Cerebral infarction (*n*, %)	13/110 (11.8%)	11/43 (25.6%)	0.035
STEMI (*n*, %)	57/110 (51.8%)	11/43 (25.6%)	0.003
cTnI (ng/mL)	1.20 (8.22)	1.77 (13.10)	0.533
CK (U/L)	179.50 (312)	212 (261)	0.585
CK-MB (U/L)	21.50 (25)	23 (30)	0.522
T-Chol (mmol/L)	4.39 ± 1.08	4.51 ± 1.16	0.561
TG (mmol/L)	1.58 (1.03)	1.68 (1.00)	0.884
HDL-c (mmol/L)	0.93 (0.32)	0.86 (0.22)	0.370
LDL-c (mmol/L)	2.67 ± 0.89	2.71 ± 0.99	0.797
ALT (U/L)	22 (17)	21 (20)	0.710
AST (U/L)	30 (34)	27 (14)	0.542
Scr (μmol/L)	72.70 (23)	105.40 (26)	< 0.001
NT-proBNP (pg/mL)	249.30 (554)	1379 (4331)	< 0.001
LVEF (%)	58 (5)	53 (11)	< 0.001
24 h mean heart rate (bpm)	66 (12)	70 (14)	0.033
SDNN (ms)	88 (38)	72 (28)	< 0.001
SDANN (ms)	74.55 ± 27.58	60.49 ± 21.97	0.003
rMSSD (ms)	25 (12)	25 (15)	0.731
pNN50 (%)	5.30 (8.30)	4.50 (8.20)	0.881
VLF (ms)	28.05 ± 12.43	19.25 ± 9.46	< 0.001
LF (ms)	14.63 (9.06)	10.28 (6.45)	0.001
HF (ms)	10.19 (6.21)	9.51 (5.28)	0.146
LF/HF	1.38 ± 0.43	1.16 ± 0.34	0.002
sHRV (*n*, %)	46/110 (41.8%)	35/43 (81.4%)	< 0.001
fQRS (*n*, %)	34/110 (30.9%)	30/43 (69.8%)	< 0.001

*AMI* acute myocardial infarction, *ALT* alanine aminotransferase, *AST* aspartate aminotransferase, *CK* creatine kinase, *CK-MB* creatine kinase isoenzyme-MB, *cTnI* cardiac troponin I, *fQRS* fragmented QRS, *HDL-c* high-density lipoprotein cholesterol, *HF* high frequency, *HRV* heart rate variability, *LDL-c* low-density lipoprotein cholesterol, *LF* low frequency, *LF/HF* ratio of low frequency to high frequency power, *LVEF* left ventricular ejection fraction, *MACE* major adverse cardiovascular events, *nMACE* non-major adverse cardiovascular events, *nsHRV* non-severely depressed heart rate variability, NT-*proBNP* N-terminal pro-brain natriuretic peptide, *pNN50* number of successive NN intervals differing by  > 50 ms divided by the total number of successive NN intervals, *rMSSD* square root of mean of the sum of squares of successive NN interval differences, *Scr* serum creatinine, *SDANN* standard deviation of all 5 min average NN intervals, *SDNN* standard deviation of NN intervals, *sHRV* severely depressed heart rate variability, *STEMI* ST-segment elevation myocardial infarction, *T-Chol* total cholesterol, *TG* triglyceride, *VLF* very low frequency

**Fig. 2:**
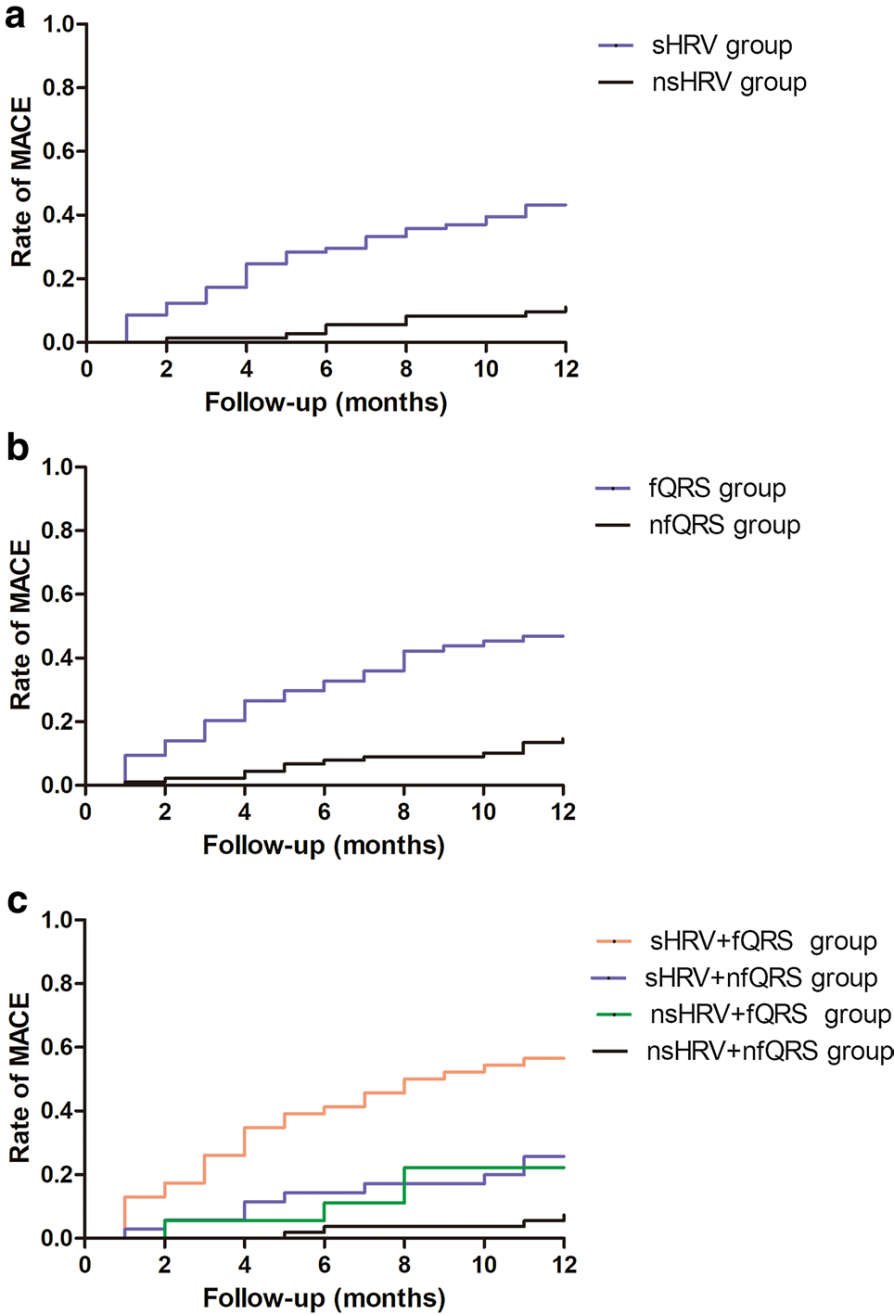
Kaplan–Meier curves comparing MACE of AMI patients with sHRV, fQRS, and combination of fQRS and sHRV. Kaplan–Meier curves demonstrated that the risk of MACE was significantly higher in the sHRV group compared to nsHRV group (log-rank test, *χ*2 = 20.422, *p* < 0.001, Fig. 2A), and in the fQRS group compared to nfQRS group (log-rank test, *χ*^2^ = 21.557, *p* < 0.001, Fig. 2B). The risk of MACE was significantly different among the four groups (log-rank test, *χ*^2^ = 36.007, *p* < 0.001). The risk of MACE was similar between the nsHRV + fQRS group and the nsHRV + nfQRS group (log-rank test, *χ*^2^ = 3.179, *p* = 0.075), and the risk of MACE was significantly higher in the sHRV + fQRS group compared to the sHRV + nfQRS group (log-rank test, *χ*^2^ = 8.376, *p* = 0.004), and sHRV + fQRS group was associated with significantly increased risk of MACE compared to nsHRV + nfQRS group (log-rank test, *χ*^2^ = 31.607, *p* < 0.001) during 12 months’ follow-up (Fig. 2C). *AMI* acute myocardial infarction, *fQRS* fragmented QRS, *HRV* heart rate variability, *MACE* major adverse cardiovascular events, *nfQRS* non-fragmented QRS, *nsHRV* non-severely depressed heart rate variability, *sHRV* severely depressed heart rate variability

During 12 months’ follow-up, Kaplan–Meier curves demonstrated that the risk of MACE was significantly higher in the sHRV group compared to the nsHRV group (log-rank test, *χ*^2^ = 20.422, *p* < 0.001) (Fig. 2A), and in the fQRS group compared to the nfQRS group (log-rank test, *χ*^2^ = 21.557, *p* < 0.001) (Fig. 2B).

The results of univariable Cox proportional hazards regression analysis showed that older age, higher Scr, NT-proBNP and 24 h mean heart rate, lower LVEF, sHRV and fQRS were risk factors of MACE, and SDNN, SDANN, VLF, LF, LF/HF values were inversely associated with MACE in AMI patients. The results of multivariable Cox proportional hazards regression analysis showed that SDNN and VLF values were inversely associated with MACE, sHRV and fQRS were independent risk factors of MACE after adjusting for age, sex, NT-proBNP, Scr and LVEF with backward elimination (Likelihood Ratio) method (Table 4).

**Table 4:** Risk factors of MACE in AMI during the 12 months’ follow-up obtained from Cox proportional hazards regression analysis

	Unadjusted HR (95% CI)	*p* value	Adjusted^*^ HR (95% CI)	*p* value
Age (yr)	1.108 (1.073–1.144)	< 0.001	–	–
Male (*n*, %)	0.622 (0.332–1.165)	0.138	–	–
Scr > 98 vs. ≤ 98 (μmol/L)	6.034 (3.240–11.235)	< 0.001	–	–
Ln NT-proBNP (pg/mL)	1.562 (1.328–1.836)	< 0.001	–	–
LVEF < 50 vs. ≥ 50 (%)	3.158 (1.664–5.992)	< 0.001	–	–
24 h mean heart rate (bpm)	1.027 (1.006–1.050)	0.013	1.018 (0.993–1.042)	0.154
SDNN (ms)	0.980 (0.969–0.991)	< 0.001	0.983 (0.969–0.998)	0.025
SDANN (ms)	0.982 (0.970–0.994)	0.004	0.990 (0.976–1.003)	0.137
VLF (ms)	0.938 (0.910−0.968)	< 0.001	0.958 (0.920–0.998)	0.038
LF (ms)	0.941 (0.896−0.987)	0.013	0.968 (0.924–1.015)	0.179
LF/HF	0.291 (0.131−0.649)	0.003	0.950 (0.388–2.325)	0.910
sHRV vs. nsHRV	4.868 (2.256−10.506)	< 0.001	2.711 (1.158–6.348)	0.022
fQRS vs. nfQRS	4.092 (2.131–7.859)	< 0.001	2.863 (1.289–6.358)	0.010

*AMI* acute myocardial infarction, *fQRS* fragmented QRS, *HR* hazard ratio, *LF* low frequency, *LF/HF* ratio of low frequency to high frequency, *Ln NT-proBNP* natural logarithm-transformed N-terminal pro-brain natriuretic peptide, *LVEF* left ventricular ejection fraction, *MACE* major adverse cardiovascular events, *nfQRS* non-fragmented QRS, *nsHRV* non-severely depressed heart rate variability, *Scr* serum creatinine, *SDANN* standard deviation of all 5 min average NN intervals, *SDNN* standard deviation of NN intervals, *sHRV* severely depressed heart rate variability, *VLF* very low frequency

*Adjusted for age, sex, Ln NT-proBNP, Scr and LVEF with backward elimination (likelihood ratio) method

The results of Chi-square test showed that there was significantly correlation between fQRS and MACE, as well as sHRV and MACE (both *p* < 0.001). The sHRV and fQRS were combined and the patients were further divided into nsHRV + nfQRS group (*n* = 54), nsHRV + fQRS group (*n* = 18), sHRV + nfQRS group (*n* = 35) and sHRV + fQRS group (*n* = 46). The incidences of MACE were 7.4, 22.2, 25.7 and 56.5%, respectively (*p* < 0.05). After adjusted for age, sex, Ln NT-proBNP, Scr and LVEF, sHRV + fQRS was an independent predictor (*p* = 0.021), using nsHRV + nfQRS as reference group, the risk of MACE was significantly increased in the other three groups, and patients in the sHRV + fQRS group had a sixfold higher risk of MACE (HR = 6.228, 95% CI 1.849–20.984, *p* = 0.003). As the whole cohort was divided into subgroups of LVEF  ≥ 50% and LVEF  < 50%, Cox regression analysis showed that sHRV + fQRS was also an independent predictor on MACE in LVEF  ≥ 50% subgroup (*p* = 0.008). Using nsHRV + nfQRS as reference group, patients in the sHRV + fQRS group had a ninefold higher risk of MACE (HR = 9.149, 95% CI 2.417–34.638, *p* = 0.001) (Table 5).

**Table 5:** Multivariate Cox regression analysis of fQRS and sHRV associated with the development of MACE in patients with AMI

	Events/total	Event rate (%)	*p* value (chi-square test)	Adjusted HR (95% CI)	*p* value
sHRV vs. nsHRV*	35/81 vs. 8/72	43.2 vs. 11.1%	< 0.001	2.140 (0.870–5.262)	0.097
fQRS vs. nfQRS**	30/64 vs. 13/89	46.9 vs. 14.6%	< 0.001	2.288 (0.993–5.268)	0.052
Combination of sHRV and fQRS***					
Whole Cohort	43/153	28.1%	< 0.001		0.021
nsHRV + nfQRS	4/54	7.4%		Reference	
nsHRV + fQRS	4/18	22.2%		6.890 (1.612–29.444)	0.009
sHRV + nfQRS	9/35	25.7%		4.108 (1.232–13.695)	0.021
sHRV + fQRS	26/46	56.5%		6.228 (1.849–20.984)	0.003
Subgroup of LVEF ≥ 50%	29/128	22.7%	0.001		0.008
nsHRV + nfQRS	4/52	7.7%		Reference	
nsHRV + fQRS	3/14	21.4%		8.235 (1.687–40.204)	0.009
sHRV + nfQRS	8/33	24.2%		3.919 (1.128–13.621)	0.032
sHRV + fQRS	14/29	48.3%		9.149 (2.417–34.638)	0.001
Subgroup of LVEF < 50%	14/25	56.0%	0.133	–	–
nsHRV + nfQRS	0/2	0%		–	–
nsHRV + fQRS	1/4	25.0%		–	–
sHRV + nfQRS	1/2	50.0%		–	–
* sHRV + fQRS*	12/17	70.6%		*–*	*–*

*AMI* acute myocardial infarction, *fQRS* fragmented QRS, *HR* hazard ratio, *HRV* heart rate variability, *LVEF* left ventricular ejection fraction, *MACE* major adverse cardiovascular events, *nfQRS* non-fragmented QRS, nsHRV, non-severely depressed heart rate variability, *sHRV* severely depressed heart rate variability

*Adjusted for age, sex, Ln NT-proBNP, Scr, LVEF and fQRS

**Adjusted for age, sex, Ln NT-proBNP, Scr, LVEF and sHRV

***Adjusted for age, sex, Ln NT-proBNP, Scr and LVEF

Kaplan–Meier curves demonstrated that the risk of MACE was significantly different among the four subgroups (log-rank test, *χ*^2^ = 36.007, *p* < 0.001). The risk of MACE was similar between the nsHRV + fQRS group and the nsHRV + nfQRS group (log-rank test, *χ*^2^ = 3.179, *p* = 0.075), and the risk of MACE was significantly higher in the sHRV + fQRS group compared to the sHRV + nfQRS group (log-rank test, *χ*^2^ = 8.376, *p* = 0.004), and the sHRV + fQRS group was associated with the highest increased risk of MACE compared to other groups (*p* < 0.001) (Fig. 2C).

### Predicting value of sHRV and fQRS for MACE in AMI patients

The predicting sensitivity, specificity and accuracy of sHRV for MACE in the AMI patients were 81.4, 58.2 and 64.7%, respectively. With fQRS as predicting parameter, the sensitivity, specificity and accuracy were 69.8, 69.1 and 69.3%, respectively. Combination of sHRV and fQRS as predicting parameters, the sensitivity, specificity and accuracy were 60.5, 81.8 and 75.8%, respectively. The specificity and accuracy were the highest with combined sHRV and fQRS as compared to sHRV or fQRS alone (Table 6).

**Table 6:** Predict value of sHRV and fQRS for MACE in AMI patients

	nMACE (*n* = 110)	MACE (*n* = 43)	Sensitivity (95% CI)	Specificity (95% CI)	PPV (95% CI)	NPV (95% CI)	Accuracy (95% CI)
sHRV	46	35	81.4 (66.6–91.6)	58.2 (48.4–67.5)	43.2 (36.9–49.7)	88.9 (80.8–93.9)	64.7 (56.6–72.3)
fQRS	34	30	69.8 (53.9–82.8)	69.1 (59.6–77.6)	46.9 (38.5–55.4)	85.4 (78.5–90.4)	69.3 (61.3–76.5)
sHRV + fQRS	20	26	60.5 (44.4–75.0)	81.8 (73.3–88.5)	56.5 (45.0–67.4)	84.1 (78.4–88.6)	75.8 (68.2–82.4)

*AMI* acute myocardial infarction, *fQRS* fragmented QRS, *HRV* heart rate variability, *MACE* major adverse cardiovascular events, *nMACE* non-major adverse cardiovascular events, *NPV* negative predictive value, *PPV* positive predictive value, *sHRV* severely depressed heart rate variability

### Incremental predictive efficacy of clinical, sHRV and fQRS in AMI patients

**Fig. 3:**
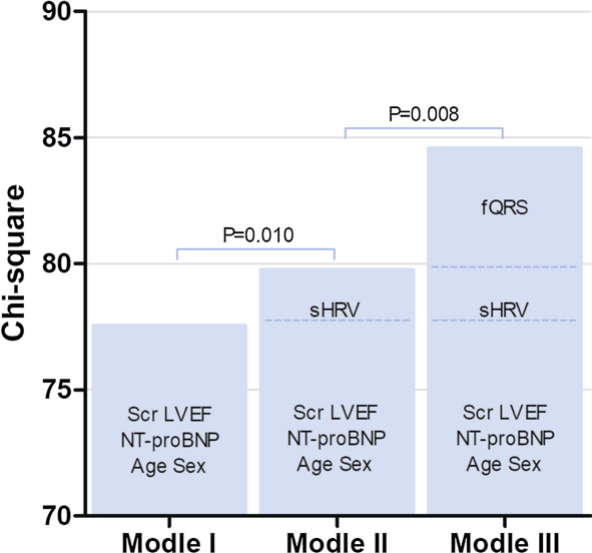
Incremental model performance for predicting prognosis assessed by starting with the clinical variables (model I: age, sex, NT-proBNP, Scr and LVEF), followed by sHRV (model II: adding sHRV to model I), and finally by adding fQRS (model III). *fQRS* fragmented QRS, *LVEF* left ventricular ejection fraction, *NT-proBNP* N-terminal pro-brain natriuretic peptide, *Scr* serum creatinine, *sHRV* severely depressed heart rate variability

Clinical variables (model I) including age, sex, NT-proBNP, Scr and LVEF were entered in the first step of a multivariable Cox model to predict MACE (Chi-square 77.54, *p* < 0.001). In model II, adding sHRV to the model I enhanced the explanatory power (Chi-square 79.56, *p* = 0.010 vs. model I). Adding fQRS (model III) further improved the prognostic performance of the model II (chi-square 84.57, *p* = 0.008 vs. model II) (Fig. 3).

## Discussion

The major findings of the present study were as follows: (1) fQRS is closely related to sHRV, suggesting the presence of significant autonomic nerve dysfunction in AMI patients with fQRS. (2) Combined assessment with fQRS and sHRV enhances the predicting efficacy on outcome in AMI patients. To the best of our knowledge, this study is the first report describing the association between fQRS and cardiac autonomic function defined by sHRV, and the prediction efficacy of combined assessment of fQRS and sHRV on outcome of patients with AMI.

### Association between fQRS and sHRV in AMI patients

The fQRS is caused by the inhomogeneous conduction of electrocardiographic activities in the infarcted and ischemic regions, reflecting the inhomogeneous electrical activity of ventricular depolarization after AMI [16]. HRV is the result of fine regulation of cardiovascular system by neurohumoral factors, which could reflect the cardiac autonomic nervous function [19, 20]. Although HRV and fQRS are essentially different in nature, fQRS and HRV could jointly hint the pathological nature in AMI patients. Our results showed that NT-proBNP was significantly higher, and LVEF was significantly lower in the fQRS group than in the nfQRS group and in the sHRV group than in the nsHRV group. The presence of fQRS might indicate more severe myocardial ischemia injury in patients with AMI, which might also lead to more severe cardiac autonomic nerve dysfunction as expressed by sHRV. The sHRV could further aggravate cardiac dysfunction in AMI patients and form vicious circle in AMI patients.

SDNN is a HRV index reflecting the total activity of the sympathetic and vagus nerves. VLF is a controversial indicator. Most studies showed that VLF was related to the sympathetic nerve [21, 22], while some study demonstrated that VLF also reflected the parasympathetic nerve activity [23]. In present study, the values of rMSSD, pNN50 and HF (all reflecting vagus nerve function) were similar between the fQRS group and the nfQRS group, while the values of SDNN and VLF were significantly lower in the fQRS group than in the nfQRS group. Our results thus hinted that there was sympathetic nerve and vagus nerve dysfunction, especially the sympathetic nerve dysfunction in AMI patients with fQRS. Previous studies showed that SDNN and VLF in HRV were the most advantageous indicators for predicting the prognosis of AMI patients [23, 24]. Therefore, SDNN and VLF, which reflect sympathetic nerve and vagus nerve function, were used to divide AMI patients into nsHRV and sHRV groups in this study.

SDNN with various cutoffs was used as a risk stratification factor in studies of MI patients. In some studies, SDNN  < 70 ms was used as a prognostic factor to predict all-cause death or cardiac death in AMI patients [11, 25]. In other studies, SDNN was classified into three categories, with SDNN  < 50 ms as a high-risk factor, and 50 ≤ SDNN < 100 ms as the moderate risk factor. SDNN  < 100 ms was considered as a moderate- to high-risk factor of defining autonomic nervous dysfunction in previous studies [17, 26, 27]. In another study, Hayano J. et al. [28] reported that the median SDNN was 90 ms for survivors and 65 ms for non-survivors in AMI group with an ejection fraction  > 35%. In our study, the median SDNN of AMI patients in the nMACE group and MACE group was 88 and 72 ms, respectively. The sensitivity, specificity, positive predictive value and negative predictive value of SDNN < 100 ms were 86.1, 36.4, 34.6 and 87.0%, respectively, and that of SDNN  < 80 ms were 65.1, 66.4, 43.1 and 83.0%, respectively, and that of SDNN < 70 ms were 44.2, 78.2, 44.2, 78.2%, respectively. The sensitivity and negative predictive value of SDNN < 100 ms as cutoff value were the highest and the incidence of missed diagnosis was the lowest. In addition, the results of multivariate Cox regression analysis showed that sHRV was an independent predictor of MACE after adjusting for age, gender, Ln-NT-proBNP, Scr and LVEF; however, with SDNN < 70 ms as cutoff value, sHRV was not an independent predictor of MACE. SDNN < 100 ms was thus used as the cutoff value in this study.

### Enhanced predicting efficacy of combined assessment in AMI patients

To verify if fQRS and sHRV were confounding factors, we performed Chi-square test and the results showed that there was significantly correlation between fQRS and MACE, as well as sHRV and MACE (both *p* < 0.001), thus fQRS and sHRV were confounding factors for the development of MACE. The predictive effect of fQRS was weakened when sHRV was added to the Cox regression model (*p* = 0.052), and the predictive effect of sHRV was weakened when fQRS was added to the Cox regression model (*p* = 0.097) (Table 5). Thus, there was interaction between fQRS and sHRV even if the correlation coefficient between fQRS and sHRV was not high (*r* = 0.322) (Table 2). Therefore, we tested the predicting value of combined sHRV and fQRS, and multivariable Cox regression analysis showed that sHRV + fQRS was an independent predictor for MACE (*p* = 0.021), and patients in the sHRV + fQRS group had a sixfold higher risk of MACE (HR = 6.228, 95% CI 1.849–20.984, *p* = 0.003). Cox regression analysis showed that sHRV + fQRS was also an independent predictor on MACE in LVEF ≥ 50% subgroup (*p* = 0.008) (Table 5). The above results indicated that coexistence of sHRV and fQRS was an alarming sign for the worse outcome in all AMI patients or AMI patients with LVEF ≥ 50%. Combined assessment with sHRV and fQRS might thus be helpful on the risk stratification of AMI patients. Individualized therapy plan should thus be applied to these patients at the highest risk. Detailed secondary preventive strategies aiming to reduce the incidence of heart failure, reinfarction, target vessel revascularization and stroke are of importance.

As expected, adding sHRV and fQRS significantly increased the predicting performance of established clinical variables including age, sex, NT-proBNP, Scr and LVEF (Fig. 3). Future clinical studies with larger patient cohort are thus warranted to validate present results and studies are also required to compare the outcome among AMI patients with sHRV and fQRS receiving usual care or intensive individualized care. Clinicians should be urged to include fQRS and sHRV in the risk stratification of AMI patients. Individualized secondary preventive strategies should be applied to patients with fQRS or sHRV, especially, patients with both fQRS and sHRV.

### Study limitations

This study is a retrospective study with a small patient cohort based on a single-center database. A larger patient cohort with multi-center database is essential to validate present results.

## Conclusion

fQRS is closely related to sHRV, suggesting significant impairment of sympathetic nerve function in AMI patients with fQRS. Combined assessment of fQRS and sHRV enhances the predicting efficacy on outcome in AMI patients.

## Supplementary Information

Below is the link to the electronic supplementary material.

Supplementary file1 (DOCX 16 kb)
