# Supplementary material for: Predicting efficacy of combined assessment with fragmented QRS and severely depressed heart rate variability on outcome of patients with acute myocardial infarction
Source: Heart Vessels. 2021 Aug 23;37(2):239–49. doi: 10.1007/s00380-021-01930-y (PMC8794902; doi:10.1007/s00380-021-01930-y)
Supplement: Supplementary file 1 — Supplementary file1 (DOCX 16 kb) [file 380_2021_1930_MOESM1_ESM.docx]

**Supplementary Table 1 Predict value of different cutoff of SDNN for MACE in AMI patients**

|  | nMACE  (n=110) | MACE  (n=43) | Sensitivity  (95% CI) | Specificity  (95% CI) | PPV  (95% CI) | NPV  (95% CI) |
| --- | --- | --- | --- | --- | --- | --- |
| SDNN<100ms | 70 | 37 | 86.1  (72.1-94.7) | 36.4  (27.4-46.1) | 34.6  (30.5-38.9) | 87.0  (75.3-93.6) |
| SDNN<80ms | 37 | 28 | 65.1  (49.1-79.0) | 66.4  (56.7-75.1) | 43.1  (35.0-51.6) | 83.0  (76.0-88.2) |
| SDNN<70ms | 24 | 19 | 44.2  (29.1-60.1) | 78.2  (69.3-85.5) | 44.2  (32.7-56.3) | 78.2  (73.0-82.6) |

AMI, acute myocardial infarction; fQRS, fragmented QRS; MACE, major adverse cardiovascular events; nMACE, non-major adverse cardiovascular events; NPV, negative predictive value; PPV, positive predictive value; SDNN, standard deviation of NN intervals.
